# CAPG Regulates Doxorubicin Resistance in Hepatocellular Carcinoma Cells via TGFB1/Smad/Nrf2 Signalling Pathway

**DOI:** 10.1111/jcmm.70847

**Published:** 2025-09-22

**Authors:** Yue Shang, Jun Zhang, Tingting Liu

**Affiliations:** ^1^ Department of Pharmacy The Second Hospital of Dalian Medical University Dalian Liaoning China; ^2^ Department of Histology and Embryology, School of Basic Medical Sciences Jilin Medical University Jilin Jilin China

**Keywords:** CAPG, chemoresistance, ferroptosis, HCC, Nrf2

## Abstract

Hepatocellular carcinoma (HCC) is a common and deadly type of liver cancer with limited treatment options and a considerable issue with chemoresistance. This study investigates the role of the cytoskeleton‐associated protein G (CAPG) gene in HCC and explores its expression, clinical relevance, as well as the molecular mechanisms on doxorubicin (Dox) resistance. Employing bioinformatics, immunohistochemistry, cell‐based assays and animal models, we systematically explored CAPG's function in HCC. Our results demonstrated that CAPG was markedly upregulated in HCC tissues and cell lines compared to normal controls (***p < 0.001). High CAPG expression was associated with poor overall survival (HR = 1.98, *p* < 0.001) and unfavourable clinicopathological parameters, indicating its potential as a prognostic biomarker. Functional experiments indicated that CAPG knockdown significantly reduced viability and proliferation in Dox‐resistant HCC cells (***p < 0.01). Conversely, overexpression promoted resistance. Mechanistically, CAPG appears to modulate ferroptosis via the TGFB1/Smad2/NRF2 signalling pathway, as supported by GSEA analysis and subsequent molecular assays. In vivo, CAPG knockdown in combination with Dox treatment significantly inhibited tumour growth in nude mouse models (***p < 0.01). These findings suggest that CAPG is a pivotal regulator of HCC progression and chemoresistance, offering a promising prognostic biomarker and combinatorial therapeutic target to overcome Dox resistance in clinical settings.

## 1 Introduction

Hepatocellular carcinoma (HCC) is one of the most prevalent and lethal malignancies in the world, presenting a considerable challenge to public health due to its high incidence and mortality rates [1, 2]. Despite progress in diagnostic and therapeutic approaches, the prognosis for HCC remains poor, primarily due to the widespread problem of chemoresistance. The emergence of resistance to chemotherapy drugs, such as doxorubicin (Dox), greatly diminishes the effectiveness of current treatment regimens, underscoring the critical need for innovative therapeutic targets and strategies [3]. In the clinical management of HCC, the development of drug resistance is a formidable obstacle to successful chemotherapy, often leading to unfavourable patient outcomes and a narrowing of treatment choices [4]. Advances in the understanding of cancer biology have revealed ferroptosis as a potential mechanism to counteract chemoresistance. This form of iron‐dependent cell death, characterised by lipid peroxidation and the buildup of reactive oxygen species (ROS), offers a novel approach to sensitise tumour cells to conventional chemotherapy agents [5]. The administration of drugs that directly modulate ferroptosis regulators or induce excessive production of lipid‐reactive ROS has demonstrated the potential to enhance the sensitivity of drug‐resistant HCC cells to treatment. A case in point was AURKA, which was identified as a suppressor of erastin‐induced ferroptosis in meningioma. AURKA interacted with KEAP1 and activated NFE2L2/NRF2, which in turn enhanced FOXM1 transcription. Suppression of AURKA in combination with erastin improved prognosis in a meningioma mouse model [6]. Phosphoenolpyruvate carboxykinase 2 (PCK2) significantly contributes to gefitinib resistance in non‐small cell lung cancer (NSCLC), making it a promising therapeutic target for enhancing treatment efficacy through modulation of ferroptosis‐related proteins, including GPX4, SLC7A11 and ASCL4 [7]. Additionally, Mitotic Checkpoint Serine/Threonine Kinase (BUB1) was highly overexpressed in PC patients, which regulated ferroptosis and contributed to gemcitabine resistance in pancreatic cancer cells, offering a potential new strategy to enhance gemcitabine efficacy [8]. Moreover, dual specificity phosphatase 4 (DUSP4) as a negative regulator of sorafenib‐induced ferroptosis in hepatocellular carcinoma (HCC) modulated key ferroptosis‐related markers and possessed potential therapeutic strategies for overcoming sorafenib resistance [9]. However, the underlying mechanism of ferroptosis in drug resistance of HCC cells remains elusive.

Recent studies have highlighted the aberrant expression of the CAPG gene in various cancers, including gastric cancer [10], triple‐negative breast cancer [11], colorectal cancer [12], Acute myeloid leukemia [13], nasopharyngeal carcinoma [14], glioblastoma [15] and bladder cancer [16], etc. implicating its role in tumour invasion and metastasis. It has been reported that CAPG is identified as a novel biomarker for early gastric cancer, contributing to tumour development through the Wnt/beta‐catenin signalling pathway [10]. Moreover, it has been highlighted the FYB1/CAPG axis as a critical determinant of AML development, offering a promising therapeutic target for acute myeloid leukaemia [17]. In colon cancer, it has been reported that CAPG interference induced cell apoptosis and ferroptosis via the P53 pathway [12]. Additionally, CAPG contributed to the progression of diffuse large B‐cell lymphoma via the PI3K/AKT signalling pathway [18]. Cumulatively, these findings underscore CAPG as a multifaceted regulator in cancer, influencing metastasis, ferroptosis, and signalling pathways across diverse malignancies. Yet, the specific function of CAPG in HCC and its potential involvement in drug resistance mechanisms remain unexplored. To address this, our study aims to uncover the precise role of CAPG in HCC progression and chemoresistance, leveraging its established ferroptosis connections to provide novel mechanistic insights. This study not only uncovers the precise role of CAPG in HCC progression and chemoresistance, but also advances the field by identifying potential diagnostic markers and therapeutic strategies for HCC.

## 2 Materials and Methods

### 2.1 Analysis of CAPG Expression in HCC and Normal Tissues

The differential expression of the CAPG gene in HCC and normal tissues was evaluated using the Xiantao platform. Specifically, CAPG gene expression levels were compared between HCC tissues and normal tissues derived from the TCGA database using the Wilcoxon rank‐sum test. Additionally, paired samples of HCC tissues (*n* = 50) and adjacent non‐cancerous tissues (*n* = 50) from the TCGA database were analysed using a paired sample *t*‐test.

### 2.2 Differential Expression Analysis Related to CAPG in HCC

Differential expression analysis of genes associated with CAPG in HCC was conducted using the Xiantao platform. Volcano plots were utilised to highlight DEGs with a threshold of |log2(FC)| > 1 and *p*.adj < 0.05. A heat map was generated to present the top 10 genes both positively and negatively correlated with CAPG expression in HCC patients.

### 2.3 Correlation of CAPG Expression With Clinicopathological Parameters

The correlation between CAPG expression and clinicopathological parameters in HCC patients was analysed using data from the TCGA database on the Xiantao platform. Based on CAPG expression levels, 535 HCC patients were categorised into high and low expression groups. Statistical analyses included the Wilcoxon rank‐sum test for continuous variables and Pearson's chi‐squared test for categorical variables. Hazard ratios were determined through univariate and multivariate analyses.

### 2.4 Survival Analysis in HCC Patients

Survival probabilities for high and low CAPG expression groups were evaluated using the Kaplan–Meier Plotter platform (www.kmplot.com) and the Xiantao platform. Subgroup survival analysis was performed based on gender, race, smoking status, and TNM stages. Hazard ratios and log‐rank *p*‐values were calculated for overall survival, disease‐specific survival and progression‐free intervals.

### 2.5 Gene Ontology (GO) and Kyoto Encyclopedia of Genes and Genomes (KEGG) Analysis and Gene Set Enrichment Analysis (GSEA) Analysis

GO and KEGG pathway analyses were conducted on CAPG co‐expressed genes using the Xiantao platform. Correlations between CAPG and other genes were analysed using the whole transcriptome gene expression data from 594 HCC patients in the TCGA project. Criteria for GO and KEGG analysis included a Spearman correlation coefficient (rho) > 0.50 and a *q*‐value < 0.05. Gene Set Enrichment Analysis (GSEA) was performed on the Xiantao platform. Initially, differential expression analysis related to CAPG in HCC patients was conducted. Subsequently, GSEA was used to elucidate significant survival differences between high‐ and low‐CAPG expression groups. Gene set permutations were performed 1000 times per analysis, with significant enrichment at FDR < 0.25 and *p*.adjust < 0.05, identifying significantly enriched signalling pathways at a false discovery rate threshold.

### 2.6 Cell Lines and Agents

The HCC cell lines HepG2 and Hep3B were obtained from ATCC (USA). These cells were cultured in Dulbecco's Modified Eagle's Medium (DMEM), supplemented with 10% fetal bovine serum (FBS, Gibco, USA) and 1% penicillin/streptomycin (#PM150110A, Procell, Wuhan, China). The cells were maintained at 37°C in a 5% CO_2_ atmosphere and 95% humidity. The Dox‐resistant variants, HepG2/Dox and Hep3B/Dox, were developed by exposing the cells to incrementally increasing concentrations of Dox over a span of 10 months. Dox (Cat. no. D1515) was purchased from Sigma‐Aldrich.

### 2.7 Transfection

Human CAPG tagged ORF clone (Cat. No. 200497) and pCMV6‐Entry (Cat. No. PS100001) plasmids were procured from OriGene Technologies Inc. (Rockville, MD, USA). Overexpression transfections were conducted using the Lipofectamine 2000 kit (Cat. No.: 11668019) from Thermo Fisher Scientific (Waltham, MA, USA). Specifically, HepG2/Dox cells were transfected with either 1 μg of pCMV6‐CAPG plasmid or 1 μg of pCMV6 plasmid in 100 μL of Opti‐MEM, following the manufacturer's protocol. Post‐transfection, cells were cultured with or without 5 μM Dox for 24 h to measure glutathione (GSH) and MDA levels.

### 2.8 Lentivirus Infection

CAPG shRNA (h) Lentiviral Particles (Cat. No. sc‐44,920‐V) and control shRNA lentiviral particles (Cat. No. sc‐108,080) were purchased from Santa Cruz Biotechnology Inc. Transfection procedures followed the kit's protocol. HepG2/Dox and Hep3B/Dox cells were transfected with Polybrene (Santa Cruz, sc‐134,220) at a final concentration of 5 μg/mL. The cell plates were gently mixed and incubated overnight at 37°C. The following day, the culture medium was replaced with 2 mL of complete medium devoid of Polybrene, and cells were incubated at 37°C for an additional 48 h in complete medium with 5% FBS. After infection, puromycin (Cat. No. sc‐108,071, Santa Cruz Biotechnology) was used to select for cells stably expressing the shRNA.

### 2.9 MTT Assay

HepG2/Dox and Hep3B cells were infected with CAPG shRNA and control shRNA lentiviruses for 24, 48, 72 and 96 h, respectively. Stable cell lines expressing CAPG shRNA and control shRNA were screened following the kit protocol. Post‐transfection, cells were subjected to 2 mg/mL puromycin for 2 weeks, and stable transfectants were validated via western blotting. Cell viability was subsequently assessed through an MTT assay, as previously described [19, 20].

### 2.10 Western Blotting Analysis

Western blotting was performed as previously described. Initially, the total protein concentration was determined and separated using 10% SDS‐PAGE. The proteins were then transferred onto a PVDF membrane and blocked with 5% skimmed milk for 1 h at room temperature. Primary antibodies used included Anti‐CAPG antibody [EPR13194‐34] (ab181095, Abcam), Nrf2 antibody (A‐10) (Cat. No. sc‐365,949, Santa Cruz Biotechnology), and β‐actin (Cat. No. ab115777, Abcam). These primary antibodies were incubated overnight at 4°C. Subsequently, the membrane was washed three times, and a secondary antibody, diluted at 1:10000, was added for 1 h at room temperature. The bands were visualised using an enhanced chemiluminescence kit (Life Technologies, USA).

### 2.11 Iron Concentration, GSH and MDA Assay

Relative levels of intracellular iron were evaluated using an iron assay kit (ab83366) as described [21]. The concentration of GSH was tested according to the kit protocol from Nanjing Jiancheng (#A006‐2). Luminescence was recorded at 405 nm and normalised to the protein concentration. MDA levels were determined using a lipid peroxidation MDA assay kit (ab118970; Abcam) by measuring cell absorbance at 532 nm.

### 2.12 Xenograft Model

For CAPG interference experiment, 6‐week‐old male BALB/c nude mice were randomly assigned to four groups: shCtrl. Group, shCAPG group, shCtrl + Dox group and shCAPG +Dox group, with 6 mice per group. For CAPG overexpression experiment, the 6‐week‐old male BALB/c nude mice were randomly assigned to three groups: untreated cell group, oe‐CAPG group and oe‐Ctrl group. A total of 2 × 10^6^ cells were inoculated into the right flank of the mice. Tumour volume and weight were monitored every 3 or 4 days. After injection, Dox (10 mg/kg) was administered orally for 14 days in both the shNC and shCAPG lentiviruses infected HepG2/Dox injected mice. Tumour volume was calculated using the formula: volume = length × width^2^ × 1/2. After 5 weeks, the mice were sacrificed, and the relevant parameters were assessed. All the experimental procedures were authorised by the Committees of Animal Ethics and Experimental Safety of The Second Affiliated Hospital of Dalian Medical University.

### 2.13 Immunohistochemistry of CAPG

The localization of the CAPG gene was examined using immunohistochemistry on tumour and adjacent normal tissues to visualise the distribution of CAPG through immunohistochemical staining. The primary antibody specific for CAPG (sc‐166,428, Santa Cruz; 1:100 dilution). The staining procedure and the assessment of staining ratios and intensities were conducted according to previously described methods [22, 23].

### 2.14 Statistical Analysis

Statistical analyses were performed using GraphPad Prism 5.0, R software (V. 4.2.1), and SPSS 13 (SPSS Inc., Chicago, IL, USA). Each experiment was conducted in triplicate. Data are presented as mean ± S.D. The differences between two groups were assessed using a two‐tailed, unpaired Student's *t*‐test. For comparisons involving multiple groups, one‐way ANOVA followed by Tukey's test was applied. A *p* value of < 0.05 was considered statistically significant.

## 3 Results

### 3.1 CAPG Is Identified as a Key Gene Associated With Dox Resistance, Prognosis in HCC, and Ferroptosis

**FIGURE 1:**
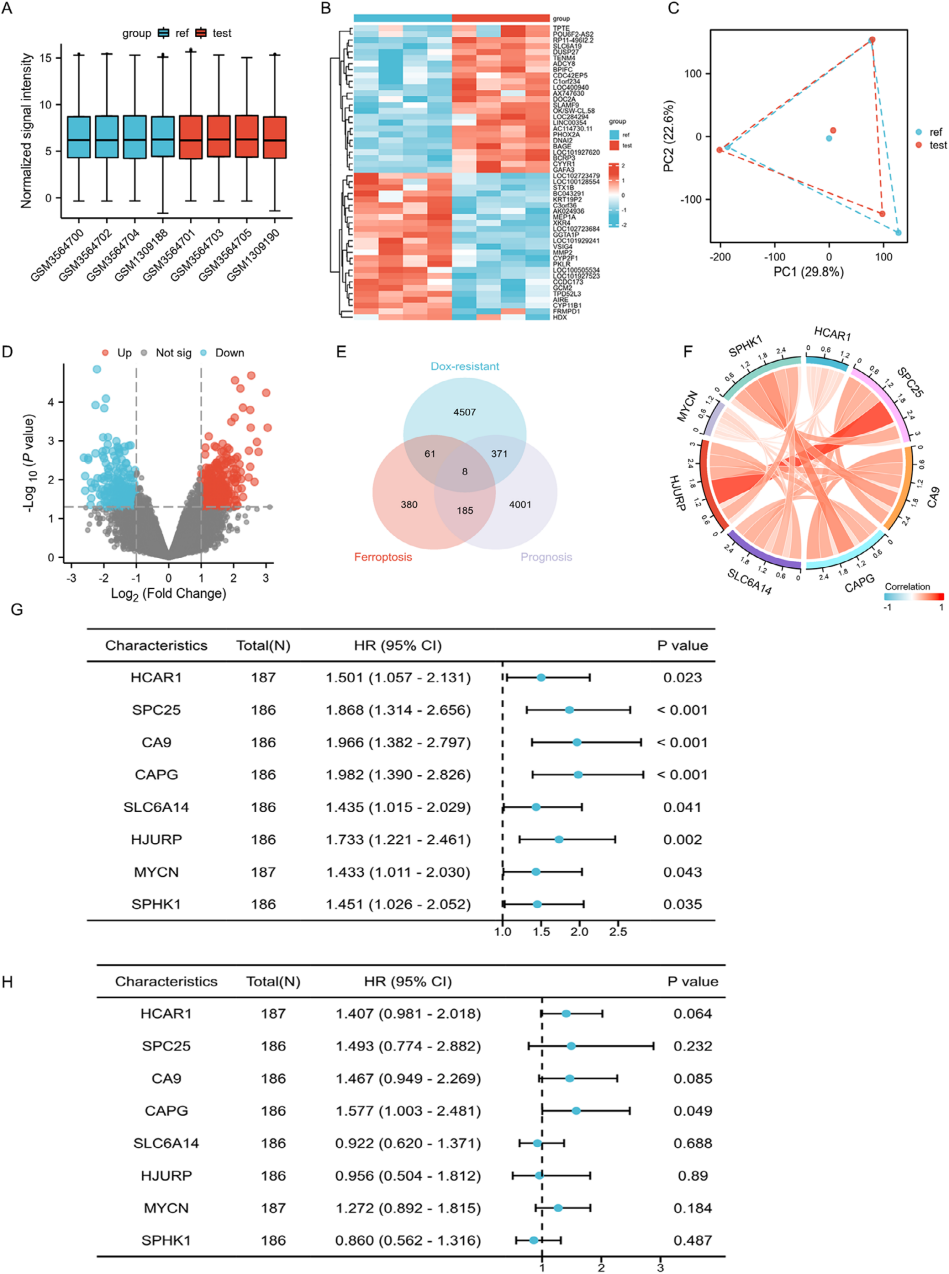
CAPG is identified as a key gene associated with Dox resistance, prognosis in hepatocellular carcinoma (HCC), and ferroptosis. (A) Box plot of sample normalisation for GSE54175 and GSE125180. (B) Heatmaps of GSE54175 and GSE125180. (C) Principal component analysis (PCA) was performed to show the differences between samples after dimensionality reduction of high‐dimensional data. (D) Volcano map was performed to show the results of differential analysis with |LogFC| > 1 and *p* value < 0.05. (E) Venn diagram was performed to show the overlapping genes among ferroptosis‐related genes, dox‐resistant genes and prognosis of HCC. (F) Chord diagram. A Spearman's rank correlation analysis was performed and visualised for eight overlapping genes from TCGA‐LIHC datasets. Univariate (G) and multivariate (H) regression analyses of the eight overlapping genes were performed to generate a forest plot.

As shown in Figure 1A–D, two datasets, GSE54175 and GSE125180, were normalised, followed by heatmap generation, principal component analysis (PCA), and volcano plot analysis, and the differentially expressed genes (DEGs) were identified. The DEGs related to Dox resistance were then crossed with the prognosis‐related gene set from liver cancer patients in the TCGA database, as well as the ferroptosis‐related gene set downloaded from the GeneCards database (with a relevance score > 1.0). A Venn diagram was performed to get 8 intersecting genes (Figure 1E). The correlations among these 8 overlapping genes were represented using a chord diagram (Figure 1F). Additionally, as shown in Figure 1G,H, univariate and multivariate regression analyses were conducted to generate a forest plot, through which CAPG was selected as the target gene for subsequent research (Table 1).

**TABLE 1:** Univariate and multivariate regression analyses were conducted for the 8 intersecting genes.

Characteristics	Total (*N*)	Univariate analysis		Multivariate analysis	
		Hazard ratio (95% CI)	*p* value	Hazard ratio (95% CI)	*p* value
HCAR1	373				
Low	186	Reference		Reference	
High	187	1.501 (1.057–2.131)	**0.023**	1.407 (0.981–2.018)	0.064
SPC25	373				
Low	187	Reference		Reference	
High	186	1.868 (1.314–2.656)	**< 0.001**	1.493 (0.774–2.882)	0.232
CA9	373				
Low	187	Reference		Reference	
High	186	1.966 (1.382–2.797)	**< 0.001**	1.467 (0.949–2.269)	0.085
CAPG	373				
Low	187	Reference		Reference	
High	186	1.982 (1.390–2.826)	**< 0.001**	1.577 (1.003–2.481)	**0.049**
SLC6A14	373				
Low	187	Reference		Reference	
High	186	1.435 (1.015–2.029)	**0.041**	0.922 (0.620–1.371)	0.688
HJURP	373				
Low	187	Reference		Reference	
High	186	1.733 (1.221–2.461)	**0.002**	0.956 (0.504–1.812)	0.890
MYCN	373				
Low	186	Reference		Reference	
High	187	1.433 (1.011–2.030)	**0.043**	1.272 (0.892–1.815)	0.184
SPHK1	373				
Low	187	Reference		Reference	
High	186	1.451 (1.026–2.052)	**0.035**	0.860 (0.562–1.316)	0.487

### 3.2 CAPG Expression Is Significantly Higher in HCC Specimens and Cell Lines

**FIGURE 2:**
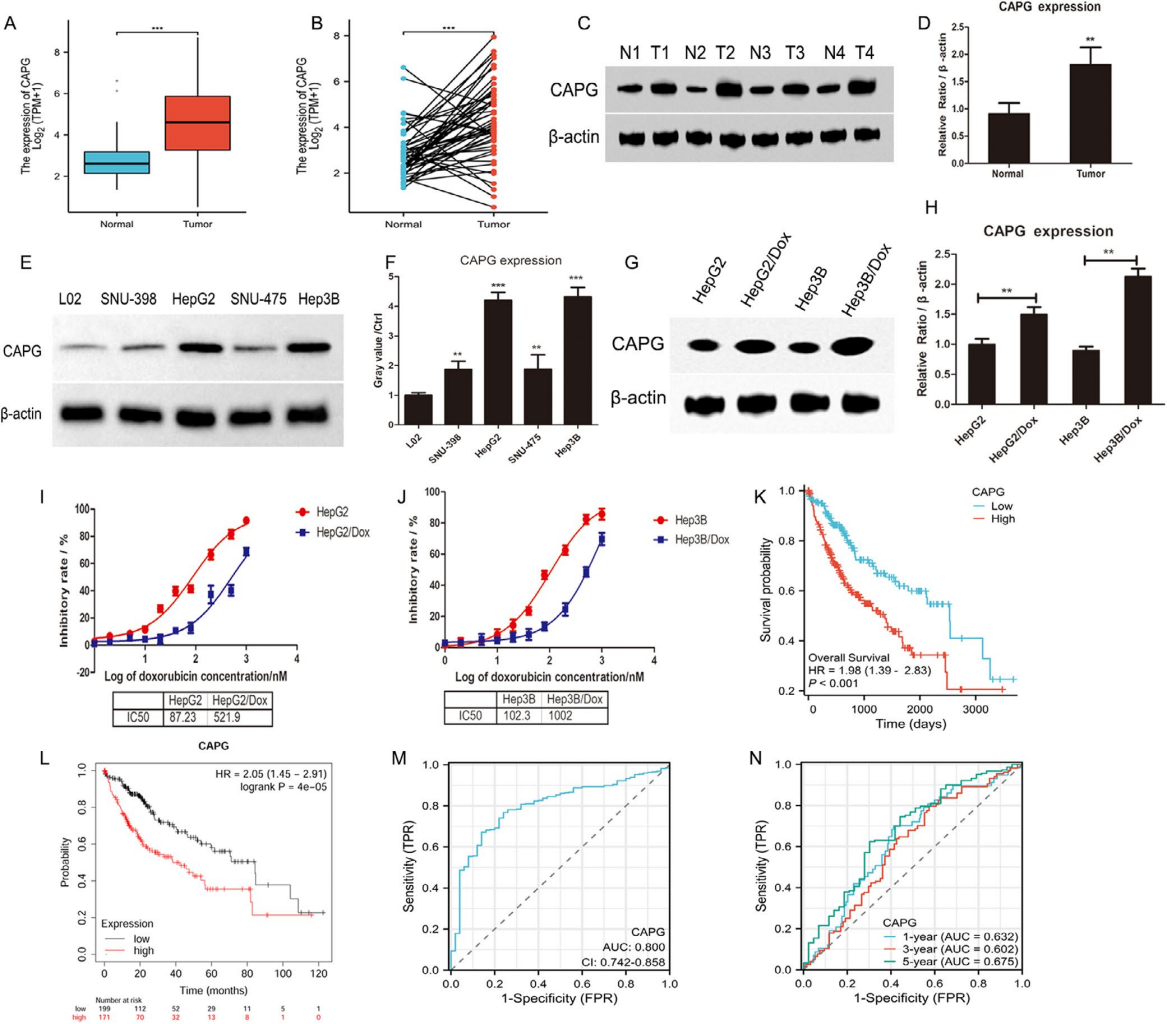
CAPG expression, diagnostic and prognostic value of CAPG in HCC patients. A. The expression of CAPG was higher in HCC tissues (*n* = 374) compared to normal tissues (*n* = 50) from the TCGA database. Wilcoxon rank sum test, ***p < 0.001. (B) CAPG showed a higher level in HCC tissues (*n* = 50) and paired non‐cancerous adjacent tissues (*n* = 50) from the TCGA database. Paired sample *t*‐test, ***p < 0.001. (C) Western blot analysis of CAPG expression in hepatocellular carcinoma (HCC) tumour tissues and normal adjacent tissues from four HCC patients. Beta‐actin was used as an internal control. (D) The quantification of CAPG expression in the tumour and control groups was shown in histograms. CAPG was significantly upregulated in HCC tumour tissues compared to their paired normal adjacent tissues. **p < 0.01, compared with the normal control group. (E) The expression of CAPG in liver cancer cell lines, including SNU‐398, HepG2, SNU‐475 and Hep3B cells. The immortal normal liver cell line L02 was used as negative control cells. (F) The relative CAPG expression in cell lines was shown in histograms. **p < 0.01, compared with that in L02 cells. (G) The expression of CAPG in HepG2/Dox, Hep3B/Dox, and their parental cell lines HepG2 and Hep3B was detected by western blotting analysis. β‐actin was used as an internal reference gene. (H) The relative CAPG was shown in the histogram. **p < 0.01, compared with the parental cell line. (I and J) The IC50 of Dox in HepG2/Dox and Hep3B/Dox was significantly higher than that in HepG2 and Hep3B, respectively. (K) Prognostic potential of CAPG in HCC patients. Overall survival (OS) was shown by the Kaplan–Meier plotter in the Xiantao platform. (L) The Kaplan–Meier Plotter database showed that high expression of CAPG in HCC patients was significantly associated with a poorer OS probability. (M) The diagnostic value of CAPG in HCC patients and normal tissues was analysed by ROC curve. (N) Time‐dependent receiver operating characteristic (ROC) curve analysis. The area under the curve (AUC) at 1 year is 0.632, at 3 years is 0.602, and at 5 years is 0.675, which showed a moderate to good predictive capability of CAPG for HCC patients.

Next, we assessed the expression of CAPG in liver cancer tissues and liver cancer cell lines. First, RNA sequencing data were downloaded from the TCGA database for the STAR process of the TCGA‐HCC (hepatocellular carcinoma) project, and data in TPM format were extracted along with clinical data of HCC patients (https://portal.gdc.cancer.gov). The results showed that CAPG expression was significantly increased in HCC tumours than that in normal controls (***p < 0.001, Figure 2A). The paired samples of normal tissues and liver cancer tissues in the TCGA database showed that CAPG expression was higher in liver cancer cells compared to normal controls (***p < 0.001, Figure 2B). Moreover, the expression of CAPG was detected and confirmed in 4 pairs of collected clinical samples. The results indicated that CAPG expression was significantly upregulated in HCC tissues compared to its paired paracancer tissues, as confirmed by Western blotting (**p < 0.01, Figure 2C,D). This was further verified by detecting the expression of CAPG in various liver cancer cell lines, including SNU‐398, HepG2, SNU‐475, and Hep3B (**p < 0.01, Figure 2E,F). The immortalised normal liver cell line L02 was used as a normal control. All the data revealed that CAPG was highly expressed in HCC tissues and potentially regulated the ferroptosis of HCC cells.

### 3.3 CAPG Contributes to Drug Resistance of Liver Cancer Cells

To further explore whether CAPG affects the drug resistance of liver cancer cells to chemotherapy agents, we established Dox‐resistant cell lines HepG2/Dox and Hep3B/Dox. As shown in Figure 2G,H, western blotting results showed that the expression level of CAPG in the resistant cell lines HepG2/Dox and Hep3B/Dox was significantly higher than that in their parental cell lines HepG2 and Hep3B (**p < 0.01). Next, we treated HepG2 and HepG2/Dox with different concentrations of Dox for 48 h, and the IC50 was determined using the MTT method. As shown in Figure 2I,J, the IC50 of Dox in HepG2/Dox was 521.9 nM, which is 5.98 times that in HepG2 (87.23 nM). Similarly, the IC50 of Dox in Hep3B/Dox was 1002 nM, which is 9.79 times that in the parental cell line Hep3B (IC50 = 102.3 nM). These results suggest that CAPG promoted the progression of liver cancer cells and enhances the resistance of liver cancer cells to Dox.

### 3.4 CAPG Is Correlated With Poor Overall Survival of Patients With HCC

Next, we explored whether CAPG correlated with the survival of patients with HCC. The relationship between CAPG expression and patient survival in HCC patients was investigated using the Kaplan–Meier plotter on the Xiantao platform and the KM plot website with the TCGA‐HCC dataset. The data showed that high expression of CAPG was significantly correlated with poor overall survival for patients with HCC in the TCGA database (HR = 1.98, *p* < 0.001) (Figure 2K,L). These results indicate that CAPG is a potential prognostic factor in HCC patients. Moreover, we assessed the prognostic value of CAPG in different subgroups. The results demonstrated that high expression of CAPG was associated with a poor prognosis in subgroups such as race (White), gender (Male), age (> 60) and pathologic stage (T3), with significant statistical significance (Figure S1). Then, the role of CAPG in HCC progression was explored by analysing the correlation between CAPG expression and clinicopathological parameters in HCC patients. As shown in Table 2, 374 cases were divided into two groups: low expression of CAPG group (*n* = 187) and high expression of CAPG group (*n* = 187). The mRNA levels of CAPG were significantly correlated with histologic grade (*p* < 0.001), histological type (*p* = 0.037), OS event (*p* = 0.035) and DSS event (*p* = 0.044). There was no significant correlation between CAPG expression and other clinicopathological factors, including age (*p* = 0.07), gender (*p* = 0.151), race (*p* = 0.427), BMI (*p* = 0.344), pathologic stage (*p* = 0.345), pathologic T stage (*p* = 0.316), pathologic N stage (*p* = 1.000), pathologic M stage (*p* = 0.146) and PFI event (*p* = 0.756). All the data indicated that CAPG promotes the progression of HCC.

**TABLE 2:** Baseline characteristics and clinical data of LIHC patients from TCGA database.

Characteristics	Low expression of CAPG	High expression of CAPG	*p* value
*n*	187	187	
Age, *n* (%)			
≤ 60	80 (21.4%)	97 (26%)	0.070
> 60	107 (28.7%)	89 (23.9%)	
Gender, *n* (%)			
Female	54 (14.4%)	67 (17.9%)	0.151
Male	133 (35.6%)	120 (32.1%)	
Race, *n* (%)			
Asian	77 (21.3%)	83 (22.9%)	0.427
Black or African American	11 (3%)	6 (1.7%)	
White	93 (25.7%)	92 (25.4%)	
BMI, *n* (%)			
≤ 25	86 (25.5%)	91 (27%)	0.344
> 25	86 (25.5%)	74 (22%)	
Histological type, *n* (%)			
Fibrolamellar carcinoma	3 (0.8%)	0 (0%)	0.037
Hepatocellular carcinoma	183 (48.9%)	181 (48.4%)	
Hepatocholangiocarcinoma (mixed)	1 (0.3%)	6 (1.6%)	
Histologic grade, *n* (%)			
G1	40 (10.8%)	15 (4.1%)	< 0.001
G2	90 (24.4%)	88 (23.8%)	
G3	49 (13.3%)	75 (20.3%)	
G4	6 (1.6%)	6 (1.6%)	
Pathologic stage, *n* (%)			
Stage I	92 (26.3%)	81 (23.1%)	0.345
Stage II	41 (11.7%)	46 (13.1%)	
Stage III	39 (11.1%)	46 (13.1%)	
Stage IV	1 (0.3%)	4 (1.1%)	
Pathologic T stage, *n* (%)			
T1	98 (26.4%)	85 (22.9%)	0.316
T2	44 (11.9%)	51 (13.7%)	
T3	38 (10.2%)	42 (11.3%)	
T4	4 (1.1%)	9 (2.4%)	
Pathologic N stage, *n* (%)			
N0	127 (49.2%)	127 (49.2%)	1.000
N1	2 (0.8%)	2 (0.8%)	
Pathologic M stage, *n* (%)			
M0	132 (48.5%)	136 (50%)	0.146
M1	0 (0%)	4 (1.5%)	
OS event, *n* (%)			
Alive	136 (36.4%)	108 (28.9%)	0.002
Dead	51 (13.6%)	79 (21.1%)	
DSS event, *n* (%)			
No	153 (41.8%)	134 (36.6%)	0.044
Yes	32 (8.7%)	47 (12.8%)	
PFI event, *n* (%)			
No	94 (25.1%)	97 (25.9%)	0.756
Yes	93 (24.9%)	90 (24.1%)	

Univariate and multivariate analyses revealed that pathologic stage, TNM stage, and histological grade were significantly associated with survival. Specifically, higher pathologic stage (II‐IV) was associated with significantly poorer survival compared to stage I (with HRs of 1.417 (0.868–2.312), *p* = 0.164; 2.734 (1.792–4.172), *p* < 0.001; 5.597 (1.726–18.148), *p* = 0.004, respectively). Larger tumour size (T2‐T4) was associated with significantly poorer survival compared to T1 (with HRs 1.431 (0.902–2.268), *p* = 0.128; 2.674 (1.761–4.060), *p* < 0.001; 5.386 (2.690–10.784), *p* < 0.001, respectively). The presence of distant metastasis (M1) significantly reduced survival compared to their respective reference groups (HRs of 4.077 (1.281–12.973), with *p* = 0.017). There were no significant associations between age, gender, race, and histological type with the survival rate of HCC patients (*p* > 0.05) (Table 3).

**TABLE 3:** Univariate and multivariable regression analysis of CAPG in liver cancer patients.

Characteristics	Total (*N*)	Univariate analysis		Multivariate analysis	
		Hazard ratio (95% CI)	*p* value	Hazard ratio (95% CI)	*p* value
Age	373				
≤ 60	177	Reference			
> 60	196	1.205 (0.850–1.708)	0.295		
Gender	373				
Female	121	Reference			
Male	252	0.793 (0.557–1.130)	0.200		
Race	361				
Asian	159	Reference			
Black or African American	17	1.585 (0.675–3.725)	0.290		
White	185	1.323 (0.909–1.928)	0.144		
Histological type	373				
Fibrolamellar carcinoma	3	Reference			
Hepatocellular carcinoma	363	3327091.5713 (0.000—Inf)	0.995		
Hepatocholangiocarcinoma (mixed)	7	2008080.3392 (0.000—Inf)	0.995		
Pathologic stage	349				
Stage I	173	Reference		Reference	
Stage II	86	1.417 (0.868–2.312)	0.164	7555945.5797 (0.000—Inf)	0.994
Stage III	85	2.734 (1.792–4.172)	**< 0.001**	4.406 (0.595–32.640)	0.147
Stage IV	5	5.597 (1.726–18.148)	**0.004**	0.000 (0.000—Inf)	0.999
Pathologic T stage	370				
T1	183	Reference		Reference	
T2	94	1.431 (0.902–2.268)	0.128	0.000 (0.000—Inf)	0.994
T3	80	2.674 (1.761–4.060)	**< 0.001**	0.727 (0.099–5.348)	0.754
T4	13	5.386 (2.690–10.784)	**< 0.001**	1.280 (0.145–11.288)	0.824
Pathologic N stage	258				
N0	254	Reference			
N1	4	2.029 (0.497–8.281)	0.324		
Pathologic M stage	272				
M0	268	Reference		Reference	
M1	4	4.077 (1.281–12.973)	**0.017**	5849002.1248 (0.000—Inf)	0.999
Histologic grade	368				
G1	55	Reference			
G2	178	1.162 (0.686–1.969)	0.576		
G3	123	1.185 (0.683–2.057)	0.545		
G4	12	1.681 (0.621–4.549)	0.307		

### 3.5 CAPG Has a High Accuracy for Diagnosis of HCC

Furthermore, the diagnostic value of CAPG expression in HCC was evaluated by ROC curve in HCC tissues and normal control tissues. The predictive value of CAPG was further analysed using a set of 374 HCC patients and 50 normal controls. As shown in Figure 2M, the specificity was 0.860 at a sensitivity of 0.67112 and the cut‐off was 3.8489. The results showed that the predictive ability of CAPG had high accuracy (AUC = 0.800, CI = 0.742–858) suggesting CAPG was a valuable diagnostic predictor in HCC patients. In assessing the prognostic value of CAPG in patients with liver cancer, we conducted a time‐dependent ROC curve analysis. The results showed the following: The AUC at 1 year was 0.632, indicating that CAPG has a moderate predictive capability for patient survival at the one‐year mark. The AUC at 3 years was 0.602, reflecting a slightly lower predictive accuracy of CAPG for survival at the 3‐year time point. The AUC at 5 years was 0.675, suggesting that CAPG's predictive capability for patient survival improves over the 5‐year period, demonstrating good predictive performance (Figure 2N). These results imply that although the predictive ability of CAPG varies at different time points, it remains a valuable biomarker for assessing the prognosis of liver cancer patients.

### 3.6 Knockdown of CAPG Overcomes Resistance to Dox in HCC Cells

**FIGURE 3:**
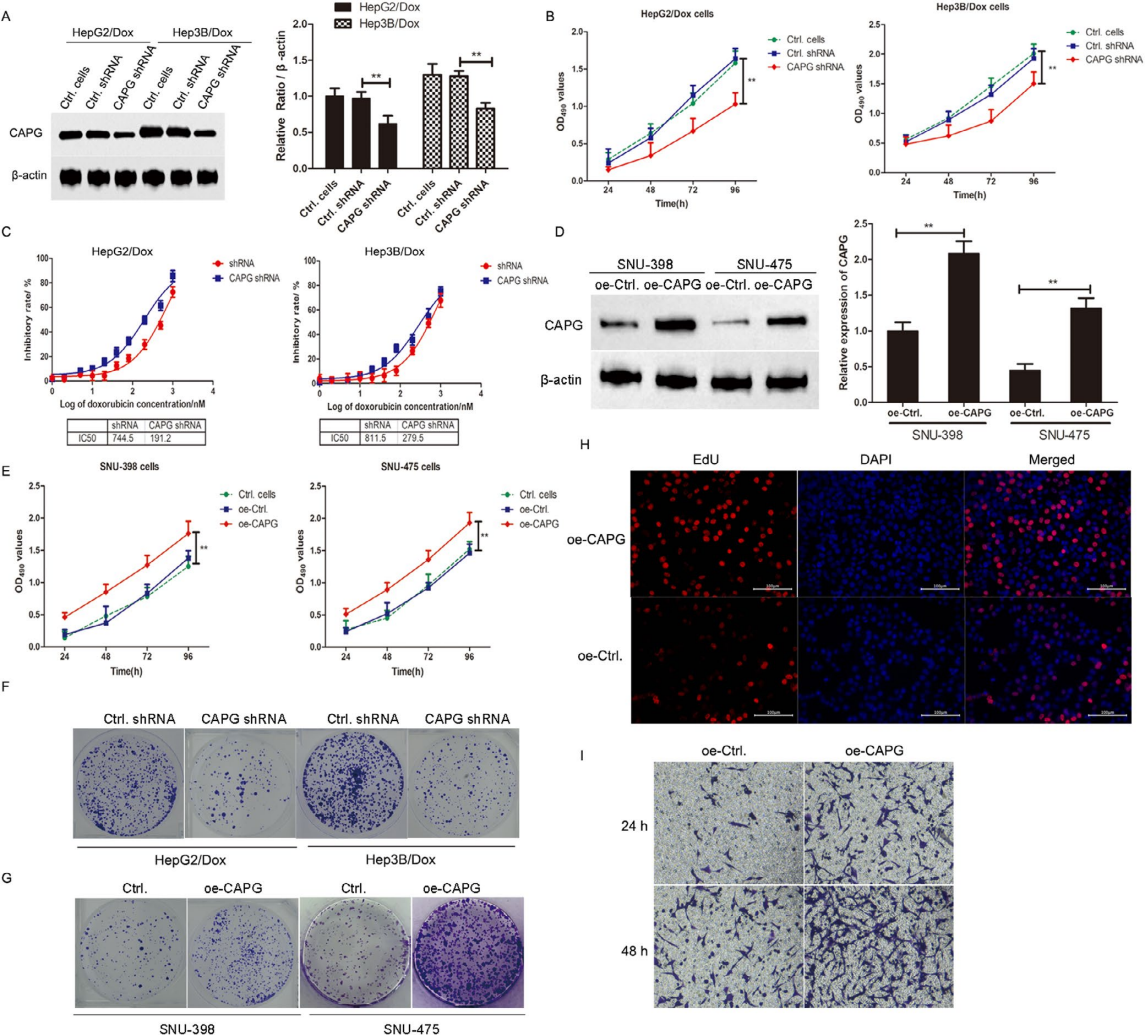
Knockdown of CAPG Overcomes Resistance to Dox in HCC Cells. (A) HepG2/Dox and Hep3B/Dox cells were infected with CAPG shRNA lentivirus or control shRNA lentivirus for 24 h. Western blotting was performed to test the expression of CAPG in untreated cells, CAPG shRNA lentivirus or control shRNA lentivirus infected cells. The relative expression of CAPG was shown in histogram. **p < 0.01, compared with control shRNA lentivirus infected cells. (B) HepG2/Dox and Hep3B/Dox cells were infected with CAPG shRNA lentivirus or control shRNA lentivirus and cultured for 24, 48, 72 and 96 h. Cell viability was determined by MTT assay. **p < 0.01, compared with shRNA lentivirus infected group. (C) HepG2, HepG2/Dox, Hep3B and Hep3B/Dox cells were treated with increasing concentrations of Dox (0~20 μM) for 48 h, and cell viability was determined by MTT assay. IC50 values were calculated by using Graphpad5.0. (D) Western blotting analysis. The expression of CAPG was determined by western blotting in oe‐CAPG lentivirus or oe‐shRNA control lentivirus infected SNU‐398 cells and SNU‐475 cells for 48 h. The relative expression of CAPG was also shown in histogram. **p < 0.01, compared with oe‐shRNA control lentivirus infected cells. (E) SNU‐398 cells and SNU‐475 cells were infected with oe‐CAPG lentivirus or oe‐control lentivirus and cultured for 24, 48, 72 and 96 h, respectively. Cell viability was determined by MTT assay. **p < 0.01, compared with oe‐control lentivirus infected group. (F) Clone formation assay. The HepG2/Dox and Hep3B/Dox cells were planted in 6‐well plate and cultured for 2 weeks in a humidified incubator at 37°C with 5% CO_2_. The medium was refreshed every 3–4 days. Colonies with more than 50 cells were counted and shown here. (G) Clone formation assay. The SNU‐398 and SNU‐475 cells were planted in 6‐well plate and cultured for 2 weeks in a humidified incubator at 37°C with 5% CO2. The medium was refreshed every 3–4 days. Colonies with more than 50 cells were counted and shown here. (H) Edu incorporation assay. Cell proliferation for the cell lines SNU‐398 cells and SNU‐475 infected by oe‐CAPG or oe‐control lentiviruses was detected by an EdU Cell Proliferation Assay Kit. SNU‐398 or SNU‐475 cells that incorporated EdU was shown in red and the nucleus was stained with DAPI. Scale: 100 μm. (I) Transwell assay. SNU‐475 cells were planted in 6‐well plate and infected with oe‐CAPG or oe‐control lentiviruses for 24 and 48 h. The images below showed the cells that have migrated or invaded through the membrane, revealing that the increased ability of oe‐CAPG cells to migrate or invade through the transwell membrane. The scale bar represents 100 μm.

In order to explore the role of CAPG in the drug resistance of HCC cells, the HCC cell lines HepG2 and Hep3B, along with their Dox‐resistant sublines HepG2/Dox and Hep3B/Dox, were used as cell models. An MTT assay was performed to test the cell viabilities of Dox‐treated HCC cells, and the results showed that after transfection with CAPG shRNA lentiviruses for 24, 48, 72 and 96 h, the cell viability of HepG2/Dox cells and Hep3B cells of shCAPG groups was significantly decreased compared with that of control shRNA groups (**p < 0.01, Figure 3A,B). To investigate the correlation between CAPG expression and Dox sensitivity, we used CAPG shRNA lentivirus to knock down CAPG in HepG2/Dox and Hep3B/Dox cells. As shown in Figure 3C, after 48 h of CAPG interference, the IC50 of Dox in HepG2/Dox cells was 191.2 nM, significantly lower than that in the shRNA virus‐infected HepG2/Dox cells (744.5 nM). Similarly, under the same conditions, the IC50 of Dox in Hep3B/Dox cells with CAPG knockdown was 279.5 nM, which was considerably lower than that in shRNA virus‐infected Hep3B/Dox cells (811.5 nM). These findings indicate that interfering with the expression of CAPG in Dox‐resistant liver cancer cells can reverse the chemotherapy resistance of these cells. In contrast, in SNU‐398 and SNU‐475 cells, overexpression of CAPG resulted in significantly higher cell proliferation efficiency compared to the negative control group (Figure 3D,E). This result was further confirmed by EdU incorporation assays (Figure 3H) and cell colony formation assays (Figure 3F,G), indicating that overexpression of CAPG promoted cell viability and proliferation efficiency in hepatocellular carcinoma cells. Furthermore, transwell assays demonstrated that overexpression of CAPG enhances cell invasion and migration (Figure 3I).

**FIGURE 4:**
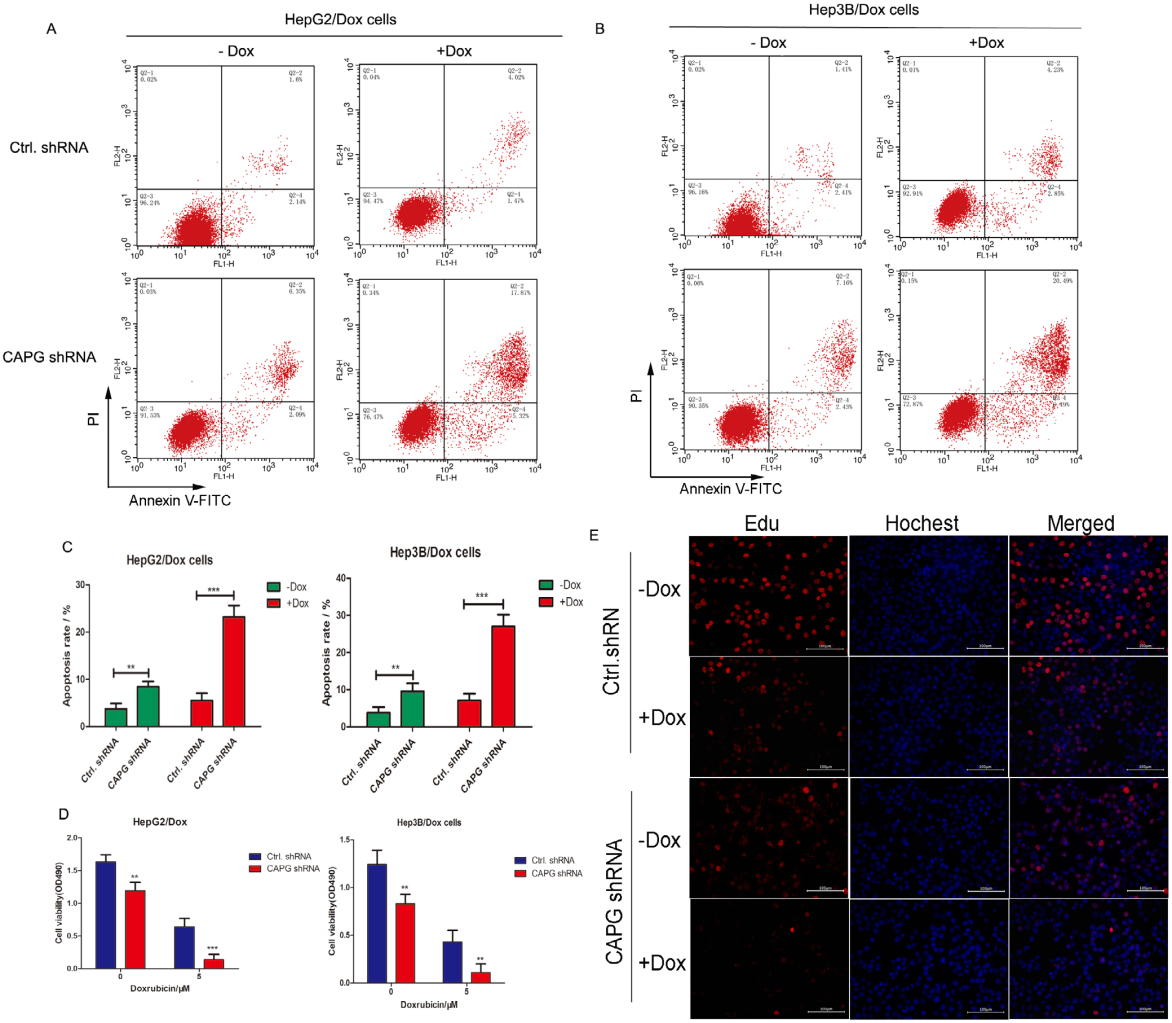
Knockdown of CAPG promotes Dox induced cell death in HepG2/Dox and ep3B/Dox cells. (A and B) The stable cell lines HepG2/Dox and Hep3B/Dox infected by shCAPG shRNA or control shRNA lentivirus were treated with 5 μM of Dox for 24 h. FACS assay was performed to test cell apoptosis rate. (C) The cell death rate determined by FACS was shown in histogram. **p < 0.01, ***p < 0.001, compared with shRNA lentivirus infected cells. (D) MTT assay. HepG2/Dox and Hep3B/Dox infected by shCAPG shRNA or control shRNA lentivirus were treated with 5 μM of Dox for 48 h. Cell viability was determined by MTT assay and shown in histogram. **p < 0.01, ***p < 0.001, compared with control shRNA group. (E) EdU incorporation assay. The stable cell lines HepG2/Dox infected by shCAPG shRNA or control shRNA lentivirus were treated with 5 μM of Dox for 24 h. Cell proliferation was detected by an EdU Cell Proliferation Assay Kit. The nucleus was stained with DAPI. Scale: 100 μM.

Furthermore, the effects of CAPG on chemotherapy drug resistance of HCC cells were tested by FACS, MTT and Edu incorporation assays. The stable cell lines HepG2/Dox and Hep3B/Dox infected by shCAPG shRNA or control shRNA lentivirus were treated with or without 5 μM of Dox for 24 h, and the results showed that interference with CAPG significantly induced the cell apoptosis rate of HepG2/Dox and Hep3B/Dox cells, as determined by the FACS assay. Moreover, the cell death rate in the CAPG shRNA interference group was significantly higher than that in the ctrl shRNA group. While interference with CAPG in combination with treatment of 5 μM Dox for 24 h further enhanced the cell death of HepG2/Dox and Hep3B/Dox (**p < 0.01, compared with Ctrl.shRNA group. Figure 4A–C). Moreover, this result was consistent with the findings from the MTT assay and Edu incorporation assay, in which the results also revealed that the knockdown of CAPG increased the Dox‐induced cell death of HepG2/Dox and Hep3B/Dox (Figure 4D,E). All the results demonstrated that the knockdown of CAPG reversed the resistance to Dox in HCC cells.

### 3.7 GO/KEGG Analysis and GSEA Analysis

**FIGURE 5:**
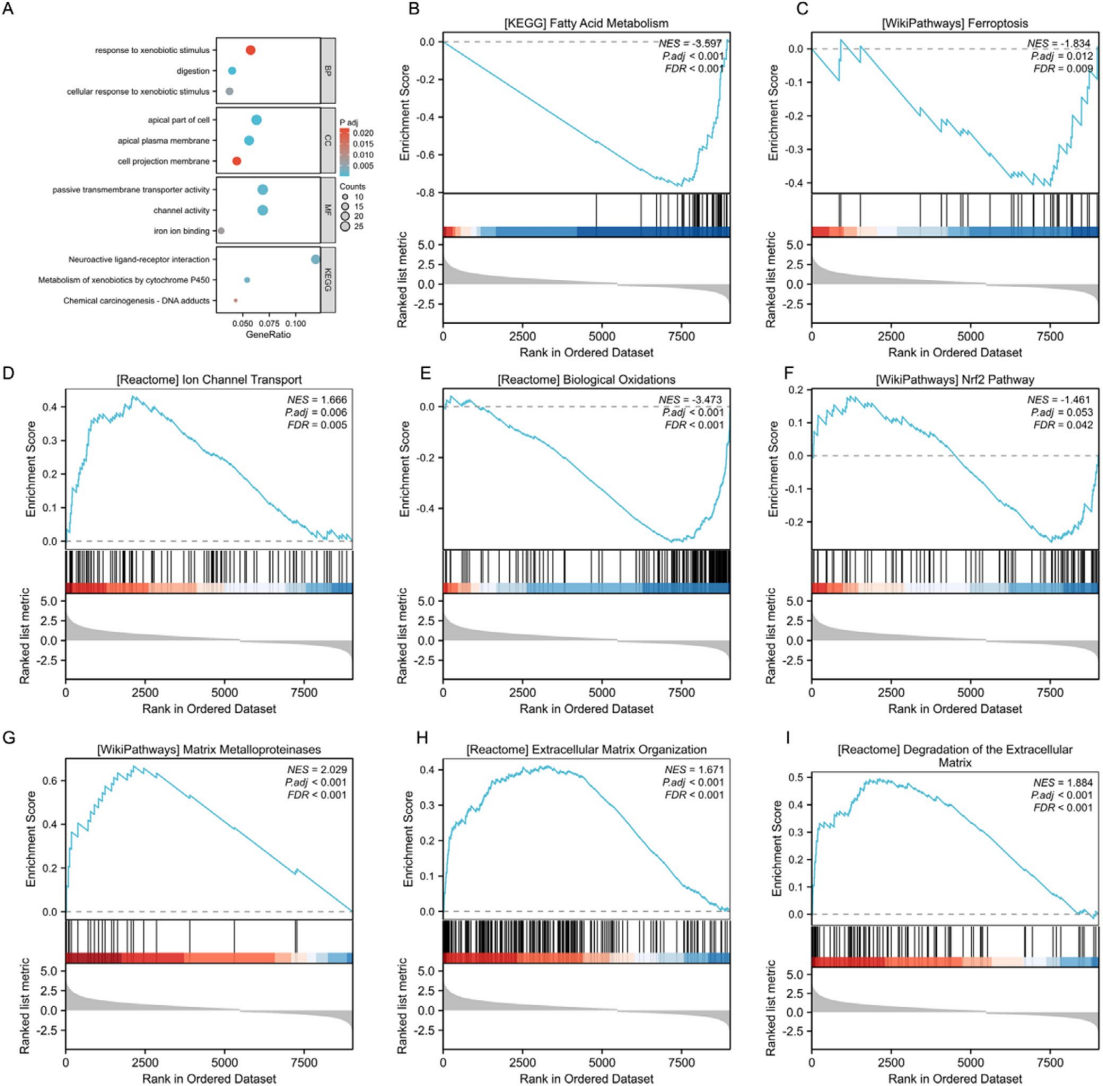
GO/KEGG analysis and GSEA analysis (A) GO/KEGG analysis. Biological process (BP), Molecular function (MF), cellular component (CC) and KEGG pathway analysis was shown. GO, Gene ontology; KEGG, Kyoto Encyclopedia of Genes and Genomes. (B–I) GSEA analysis.

Furthermore, we explored the anticancer molecular mechanisms and associated signalling pathways of CAPG in hepatocellular carcinoma cells. GO/KEGG analysis revealed that CAPG was associated with the following molecular functions: passive transmembrane transporter activity, channel activity, and iron ion binding (Figure 5A). GSEA assays indicated that CAPG was related to pathways including fatty acid metabolism, ferroptosis, ion channel transport, biological oxidations, the NRF2 pathway, matrix metalloproteinases, extracellular matrix organisation, and degradation of the extracellular matrix (Figure 5B–I). These results suggest that CAPG may be involved in the processes of ferroptosis as well as cell infiltration and migration during the development of drug resistance in hepatocellular carcinoma (HCC).

### 3.8 Knockdown of CAPG Promotes Erastin and Dox‐Induced Ferroptosis in HepG2/Dox and Hep3B/Dox Cells

**FIGURE 6:**
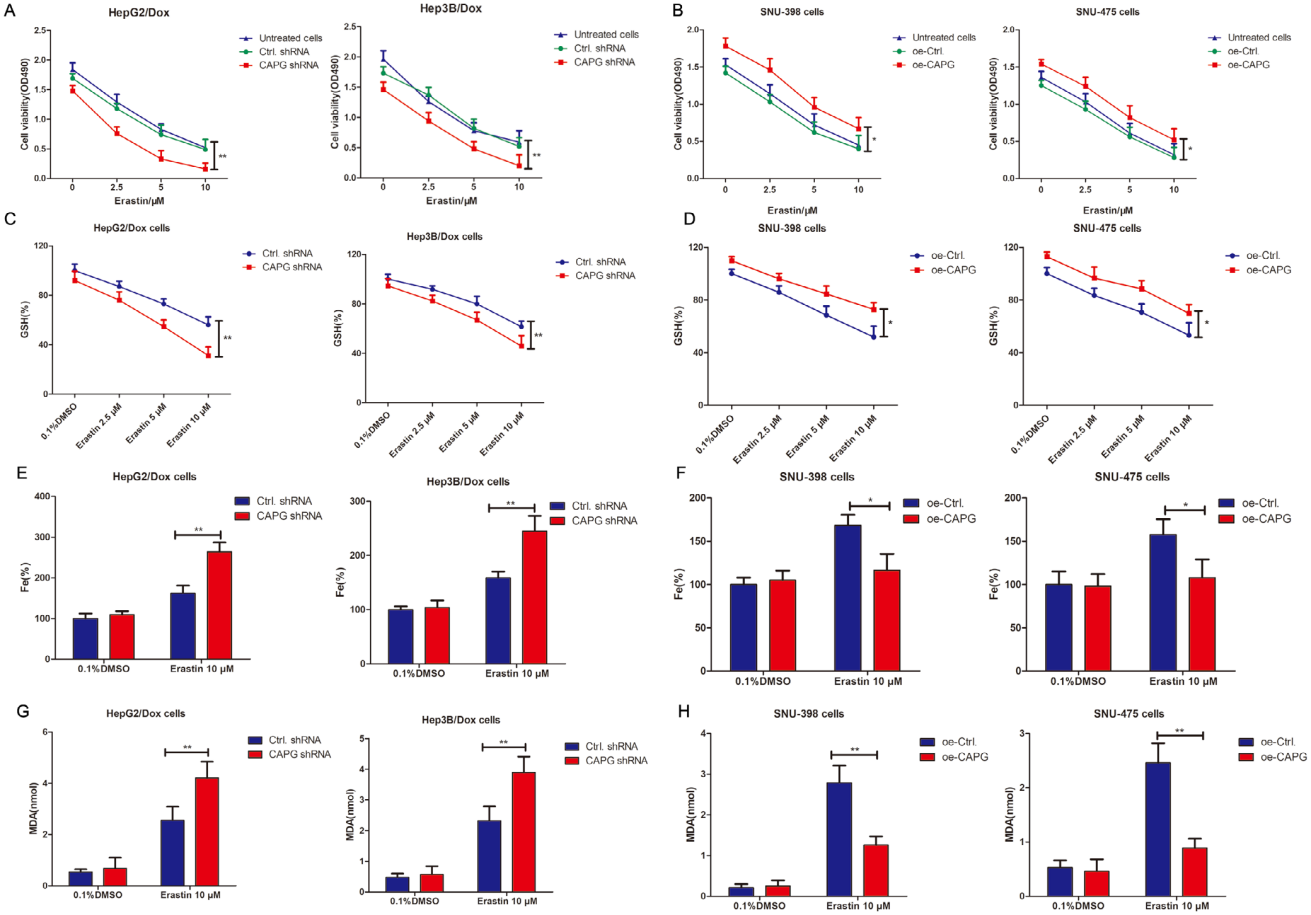
Knockdown of CAPG promotes erastin‐induced ferroptosis in HepG2/Dox and Hep3B/Dox Cells. (A) The CAPG shRNA or control shRNA lentiviruses infected HepG2/Dox and Hep3B/Dox cells were treated with increasing concentrations of erastin, including 2.5, 5 and 10 μM for 48 h. Cell viability was performed by MTT assay. **p < 0.01, compared with control shRNA group. (B) SNU‐398 and SNU‐475 cells were infected with oe‐CAPG or oe‐Ctrl lentiviruses and treated with increasing doses of erastin at the concentrations of 2.5, 5 and 10 μM for 48 h. MTT assay was performed to test the cell viability of each group. *p < 0.05, compared with oe‐control group. (C) The shCAPG or control shRNA lentiviruses infected HepG2/Dox and Hep3B/Dox cells were treated with increasing concentration of erastin (2.5, 5 and 10 μM) for 48 h and the relative GSH levels were tested according to kit protocol. **p < 0.01, compared with control group. (D) The oe‐CAPG or oe‐control lentiviruses infected SNU‐398 and SNU‐475 cells were treated with increasing concentration of erastin (2.5, 5 and 10 μM) for 48 h and the relative GSH levels were tested according to kit protocol. *p < 0.05, compared with oe‐control group. (E) shCAPG shRNA or control shRNA lentiviruses infected HepG2/Dox or Hep3B/Dox cells were treated with 10 μM of erastin for 48 h. The relative Fe levels were tested according to kit protocol. **p < 0.01, compared with control group. (F) The oe‐CAPG or oe‐control lentiviruses infected SNU‐398 and SNU‐475 cells were treated with 10 μM of erastin for 48 h. The relative Fe levels were tested according to kit protocol. *p < 0.05, compared with control group. (G) The MDA level was tested in shCAPG or control shRNA lentiviruses infected HepG2/Dox and Hep3B/Dox after being treated with or without 10 μM of erastin for 48 h. **p < 0.01, compared with control group. (H) The MDA level was tested in oe‐CAPG or oe‐control lentiviruses infected SNU‐398 and SNU‐475 cells after being treated with or without 10 μM of erastin for 48 h. **p < 0.01, compared with control group.

In order to determine whether the cell death of HepG2/Dox and Hep3B/Dox cells was associated with ferroptosis, we treated these cells with the ferroptosis activator erastin or chemotherapeutic drugs such as Dox. Interference with CAPG promoted cell death induced by erastin in HepG2/Dox and Hep3B/Dox cells, and the cell death increased in a dose‐dependent manner with the concentration of erastin (Figure 6A). Moreover, cell death induced by erastin was inhibited in oe‐CAPG lentivirus‐infected SNU‐398 and SNU‐475 cells compared to cells infected with Ctrl shRNA lentiviruses after 48 h (Figure 6B). Furthermore, GSH levels were significantly suppressed in a dose‐dependent manner in erastin‐treated HepG2/Dox and Hep3B/Dox cells, while GSH levels were significantly increased after treating oe‐CAPG transfected SNU‐398 and SNU‐475 cells with erastin for 48 h, compared with control groups (Figure 6C,D). Additionally, iron ion and MDA concentrations were significantly increased in CAPG shRNA‐transfected HepG2/Dox and Hep3B/Dox cells treated with 10 μM erastin, whereas overexpression of CAPG inhibited the concentrations of Fe and MDA (Figure 6E–H).

**FIGURE 7:**
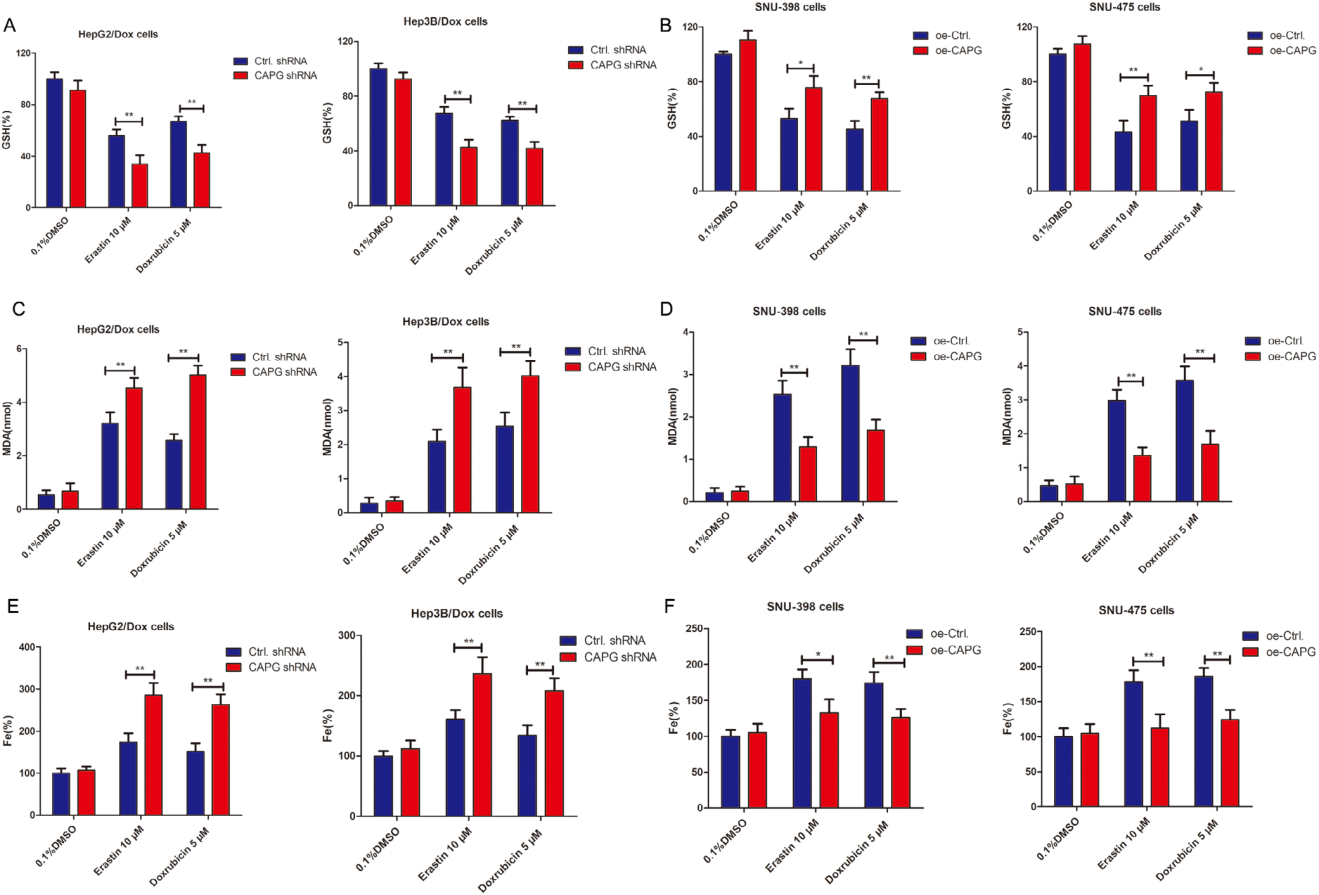
Knockdown of CAPG promotes erastin and Dox‐induced ferroptosis in HepG2/Dox and Hep3B/Dox cells. (A and B) The relative GSH levels. The shCAPG or control shRNA lentiviruses infected HepG2/Dox and Hep3B/Dox cells, oe‐CAPG or oe‐control lentiviruses infected SNU‐398 and SNU‐475 cells were treated with 10 μM of erastin or 5 μM of Dox for 48 h. The relative GSH levels were tested according to kit protocols. *p < 0.05, **p < 0.01, compared with the control group. (C and D) The shCAPG or control shRNA lentiviruses infected HepG2/Dox and Hep3B/Dox cells, oe‐CAPG or oe‐control lentiviruses infected SNU‐398 and SNU‐475 cells were treated with 10 μM of erastin or 5 μM of Dox for 48 h. The MDA levels were tested according to kit protocols. *p < 0.05, **p < 0.01, compared with the control group. (E and F) Fe^2+^ relative levels. The shCAPG or control shRNA lentiviruses infected HepG2/Dox and Hep3B/Dox cells, oe‐CAPG or oe‐control lentiviruses infected SNU‐398 and SNU‐475 cells were treated with 10 μM of erastin or 5 μM of Dox for 48 h. The MDA levels were tested according to kit protocols. *p < 0.05, **p < 0.01, compared with the control group.

Furthermore, we tested whether Dox induced ferroptosis of drug‐resistant HCC cells. As shown in Figure 7A, intracellular GSH was significantly decreased in sh‐CAPG lentivirus infected cells compared to the NC group following erastin (10 μM, 24 h) or Dox (5 μM, 24 h) administration. While MDA, the end product of lipid peroxidation, was remarkably increased in sh‐CAPG lentivirus infected cells, as well as iron levels. Correspondingly, in oe‐CAPG transfected cells, levels of MDA and iron were markedly decreased, while GSH was increased, compared with the NC group (*p < 0.05, **p < 0.01, Figure 7B–F).

### 3.9 Knockdown of CAPG Increased Ferroptosis Induced by Dox via TGFB1/Smad/Nrf2 Signaling Pathway

**FIGURE 8:**
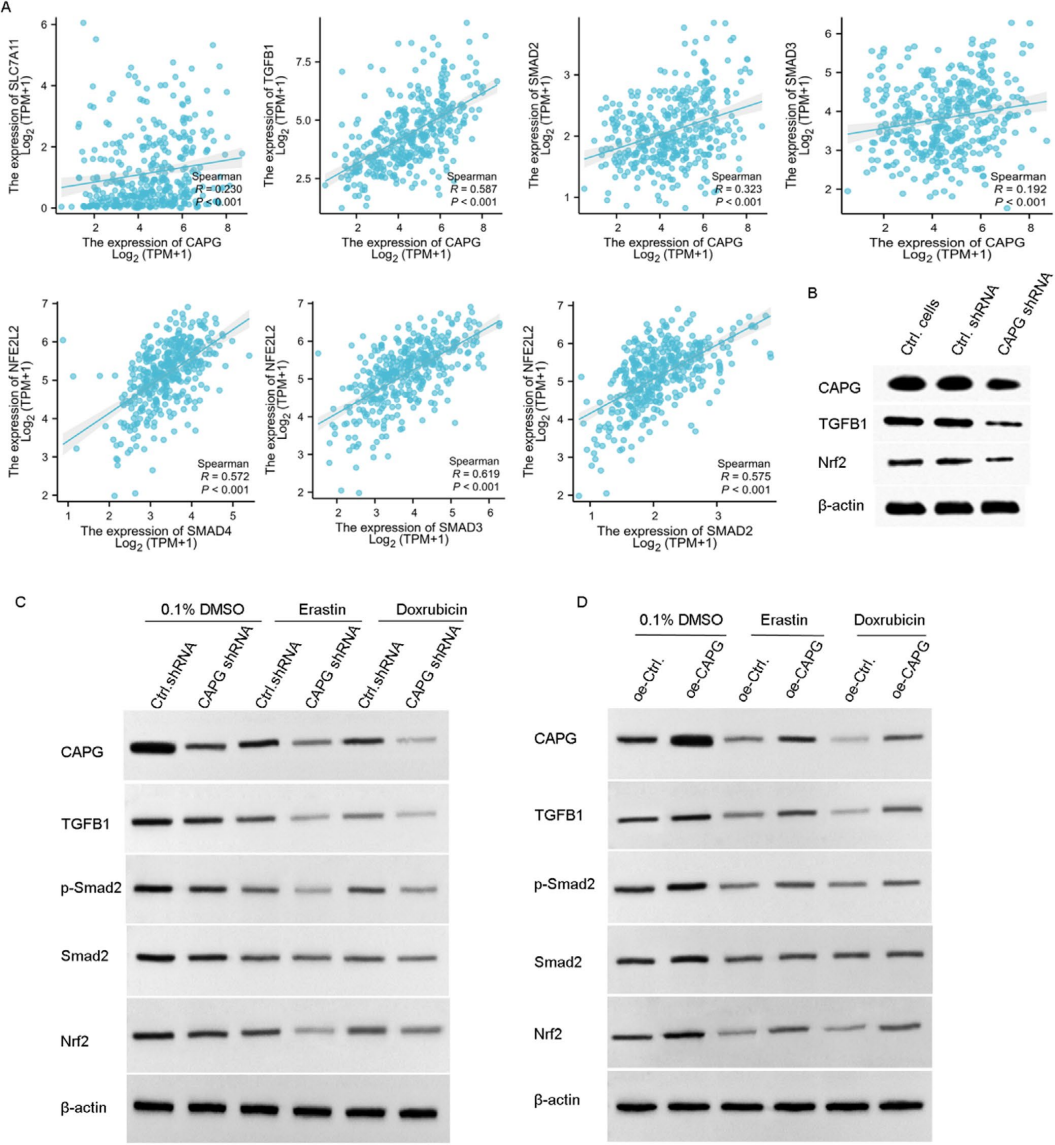
Knockdown of CAPG increased ferroptosis induced by Dox via TGFB1/Smad/Nrf2 signalling pathway. (A) RNA‐seq data was downloaded from the TCGA‐LIHC (Liver Hepatocellular Carcinoma) project in TCGA database (https://portal.gdc.cancer.gov) using the STAR alignment method. The data in TPM (Transcripts Per Million) format was downloaded and the clinical data were also downloaded. The correlation between the expression level (TPM) of CAPG and SLC7A11, TGFB1, SMAD2/3/7, as well as Nrf2 and Smad2/3. (B) Hep3B/Dox cells were infected with CAPG shRNA or Ctrl. shRNA lentiviruses and co‐treated with 10 μM of Dox for 24 h, the expression of CAPG, TGFB1, Nrf2 was detected by western blotting. (C) HepG2/Dox cells were infected with CAPG shRNA or Ctrl. shRNA lentiviruses and co‐treated with 10 μM of erastin or 5 μM of Dox for 48 h; the expression of CAPG, TGFB1, p‐smad2, smad2 and Nrf2 was detected by western blotting. (D) SNU‐475 cells were infected with oe‐CAPG or oe‐control lentiviruses and co‐treated with 10 μM of erastin or 5 μM of Dox for 48 h; the expression of CAPG, TGFB1, p‐smad2, smad2 and Nrf2 was detected by western blotting.

The drug resistance of Dox usually appeared during therapy; we have found that knockdown CAPG reversed the drug resistance of Dox in HCC cells, possibly related to ferroptosis. Next, the related molecular mechanism of CAPG in ferroptosis of HCC cells was explored. It has been found that GSEA analysis showed CAPG was related to the Nrf2 axis in hepatocellular carcinoma cells. Firstly, TCGA‐HCC RNAseq data was downloaded from the TCGA database (https://portal.gdc.cancer.gov), and the correlation of CAPG and TGFB1 or smad2/3 in HCC patients was analysed. As shown in Figure 8A, Spearman analysis was performed and the correlation coefficient R was 0.587, 0.1323, 0.132, respectively, indicating CAPG and tgfb1, smad2/3 have a positive correlation. Moreover, the Nrf2 had a positive correlation with Smad2 (*R* = 0.575, *p* < 0.001), Smad3 (*R* = 0.619, *p* < 0.001) and Smad4 (*R* = 0.572, *p* < 0.001), respectively (Figure 8A).

Additionally, the expression of CAPG, TGFB1 and Nrf2 was tested in HEPG2/Dox cells, and the results showed that TGFB1 and Nrf2 were obviously decreased in CAPG shRNA lentivirus infected HEPG2/Dox cells (Figure 8B). Moreover, the Hep3B cells were infected with CAPG shRNA lentivirus and control shRNA lentivirus for 24 h, with or without incubating with 10 μM of erastin or Dox (5 μM). The results showed that the expression of TGFB1, p‐smad2/3 and Nrf2 were significantly decreased in erastin or Dox treated cells than that in the cells without the drugs treated cells (**p < 0.01, Figure 8C). While the stable cell lines of SNU‐375 transfected with pCMV6‐CAPG or pCMV6 vector were treated with or without Dox (5 μM) for 24 h. The results showed that overexpression of CAPG promoted the expression of TGFB1, p‐SMAD2/3, and Nrf2 in SNU‐375 cells compared to that in oe‐Ctrl transfected cells (**p < 0.01). Levels of TGFB1, p‐SMAD2/3, and Nrf2 were decreased in erastin or Dox‐treated CAPG shRNA‐transfected SNU‐375 cells compared to non‐drug treated cells (Figure 8D). Overall, these results indicate that the knockdown of CAPG reverses Dox resistance by inducing ferroptosis through the TGFB1/Nrf2 signalling pathway in hepatocellular carcinoma (HCC) cells.

### 3.10 CAPG Promotes HCC Tumour Progression in Nude Mice

**FIGURE 9:**
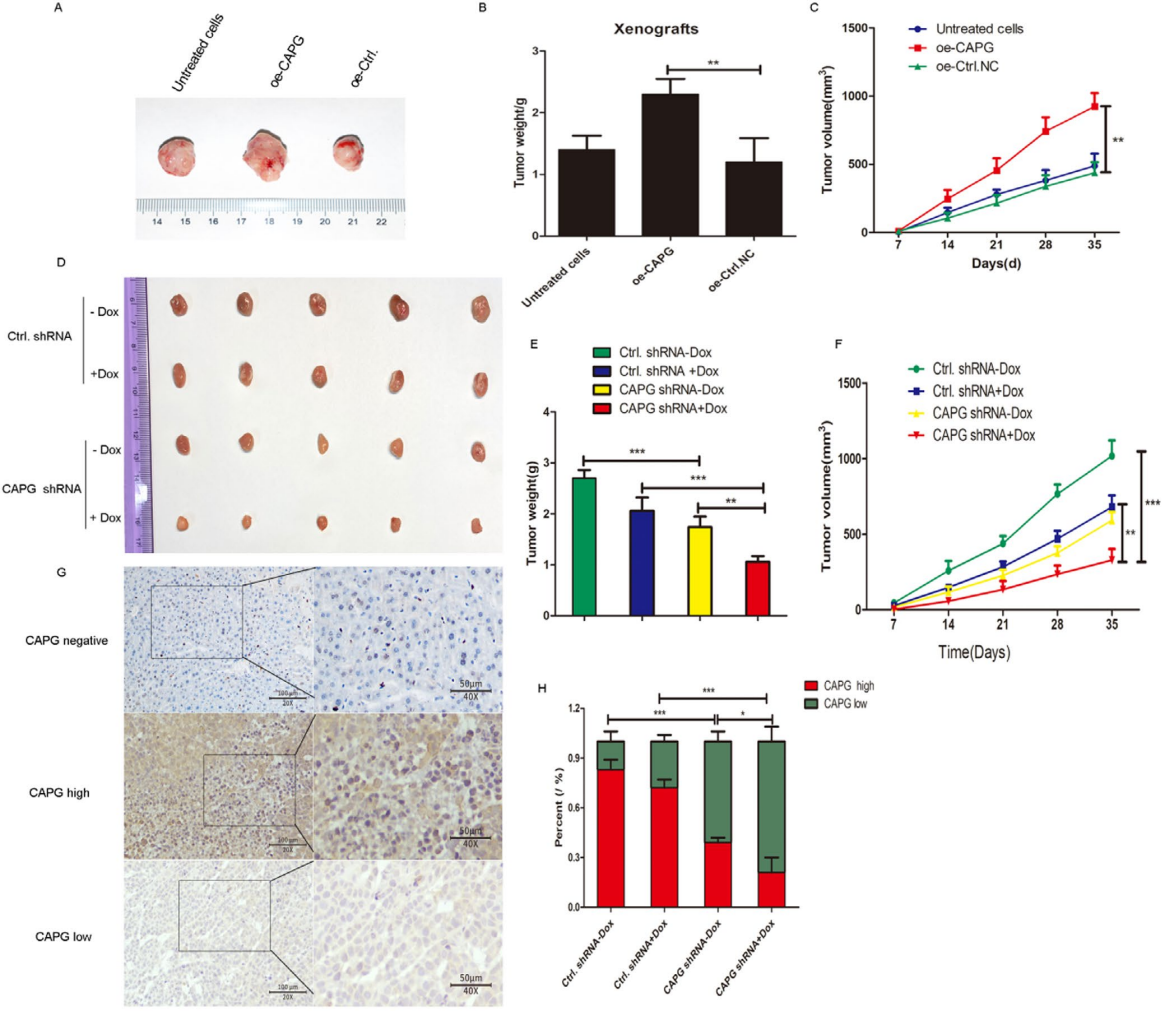
CAPG promotes HCC tumour progression in nude mice. For the overexpression experiment, 100 μL of 3 × 10^7^ cells/mL SNU‐475 cells infected with oe‐Ctrl or oe‐CAPG lentiviruses, and untreated SNU‐475 cells were subcutaneously injected into the right dorsal flanks of C57BL/6 mice (*n* = 6 for each group). Mice were sacrificed 5 weeks after injection. Representative tumour images (A), tumour weight (B) and tumour volume (C) were shown in each group. **p < 0.01, compared with the oe‐control group. In the other experiment, 100 μL of 3 × 10^7^ cells/mL stable HepG2/Dox cells infected with shCtrl or shCAPG lentiviruses were subcutaneously injected into nude mice (*n* = 6). One week later, the shCtrl or shCAPG group was administered orally with or without Dox (10 mg/kg) for 14 days. Representative tumour images (D), tumour weight (E), tumour volume (F), (G) immunohistochemical staining for CAPG were shown in each group. (H) The percent of CAPG expression was shown in a histogram in each group. *p < 0.05, **p < 0.01, ***p < 0.001, compared with the control group.

In order to test the effects of CAPG in vivo, nude mice were subcutaneously injected with SNU‐475 cells infected with oe‐Ctrl or oe‐CAPG lentiviruses (100 μL of 3 × 10^7^ cells/mL SNU‐475 cells) to validate the oncogenic activity of CAPG. The animal experiment results showed that in nude mice subcutaneously inoculated with SNU‐475 cells overexpressing CAPG and oe‐Ctrl, tumour volume was measured every 3–4 days. After 5 weeks, the mice were sacrificed, and tumours were excised and photographed. As shown in Figure 9A–C, the tumour weight in the oe‐CAPG group was significantly greater than that in the control group. Furthermore, the tumour volume in the oe‐CAPG group was significantly larger than that in the oe‐Ctrl group and the control cell group (**p < 0.01). These findings indicated that overexpression of CAPG promotes tumour progression in mice.

On the contrary, nude mice were subcutaneously injected with stable HepG2/Dox cells infected with shCtrl or shCAPG lentiviruses (100 μL of 3 × 10^7^ cells/mL HepG2/Dox) to validate the oncogenic activity of CAPG. Tumour volumes were measured every 3 or 4 days. One week later, the shCtrl or shCAPG group was administered orally with or without Dox (10 mg/kg) for 14 days. After 5 weeks, the mice were sacrificed, and four images of tumours from each group were captured. The results indicated that interference with CAPG significantly inhibited tumour growth in vivo when combined with Dox (10 mg/kg) (Figure 9D). Tumour volume and tumour weight were significantly reduced in the shCAPG lentivirus group, both with and without Dox treatment, compared to the shRNA lentivirus group (**p < 0.01, ***p < 0.001, Figure 9E,F). Moreover, immunohistochemical analysis showed that the protein levels of CAPG were markedly decreased in the shCAPG lentivirus‐infected group treated with Dox compared with the controls (Figure 9G,H). Thus, knockdown of CAPG clearly inhibited the progression of HCC.

## 4 Discussion

Despite advancements in diagnostic and therapeutic strategies, the prognosis for HCC patients remains poor, particularly due to the issue of chemoresistance. Understanding the molecular mechanisms underlying HCC progression and drug resistance is crucial for developing more effective treatments. The CAPG gene, known for its aberrant expression in various cancers and its association with tumour invasion and metastasis, has emerged as a potential player in HCC. Although it has been reported that CAPG was expressed in the cytoplasm of hepatocellular carcinoma cells and was associated with metastasis and poor prognosis, suggesting its potential as a diagnostic biomarker [24]. However, its specific role in HCC and its impact on drug resistance remain unclear, necessitating further investigation. Our study clarified the role of CAPG in HCC, particularly its expression patterns, association with clinical outcomes, and involvement in chemoresistance. Utilising a combination of bioinformatics analysis, cellular experiments, and animal models, we systematically explored CAPG's function in HCC. Key findings include the high expression of CAPG in HCC tissues, its correlation with poor patient prognosis and clinical parameters, and its role in modulating Dox resistance. These results suggest that CAPG could serve as a valuable biomarker and therapeutic target in HCC, offering new avenues for improving patient outcomes.

The findings of this study elucidate the significant role of CAPG in HCC through its involvement in molecular mechanisms and signalling pathways. The GSEA analysis and subsequent western blot assays indicated that CAPG was involved in regulating HCC cell survival and proliferation by modulating the Nrf2 signalling pathway, which is known to play a role in cellular defence against oxidative stress and ferroptosis [25, 26, 27]. The interference with CAPG expression promoted Dox‐induced ferroptosis, revealing the potential mechanism by which CAPG contributes to chemoresistance in HCC. These results provide a deeper understanding of the molecular underpinnings of HCC and suggest that targeting CAPG could be a viable strategy for overcoming drug resistance in HCC therapy.

Despite established roles of CAPG in promoting metastasis and ferroptosis in other cancers (e.g., gastric cancer via Wnt/β‐catenin [10], colorectal cancer via p53 [12]), its specific function in HCC–particularly in chemoresistance mechanisms–remains a critical unexplored gap. Our work directly addresses this void by defining CAPG‐driven ferroptosis regulation as a novel therapeutic target, thereby advancing HCC treatment strategies beyond prior pan‐cancer studies. In terms of gene function and cellular behaviour, the study demonstrates that CAPG significantly influences the proliferation and viability of HCC cells. The knockdown of CAPG in Dox‐resistant HCC cell lines resulted in decreased cell viability and increased sensitivity to Dox, indicating that CAPG plays a crucial role in maintaining the drug‐resistant phenotype. Specifically, in the present study, the correlation between CAPG and the TGFB1/Smad/Nrf2 signalling pathway suggests that CAPG may influence the proliferation and metastasis of HCC. The TGFB1 pathway is known to play a role in immune suppression and tumour progression. The findings that CAPG knockdown led to decreased levels of TGFB1 and its downstream effectors, Smad2/3, indicate that CAPG may contribute to Dox resistance via the TGFB1/Smad2/3/Nrf2 signalling pathway in HCC cells. This is further supported by the increased ferroptosis observed in CAPG knockdown cells, which could enhance the sensitivity of drug‐resistant HCC cells and reverse the drug resistance response. These results underscore the potential of targeting CAPG not only to inhibit tumour growth and metastasis but also to reverse chemotherapeutic drug resistance in HCC, providing a dual approach to cancer therapy.

Finally, despite the robust statistical significance observed in our bioinformatics and in vitro analyses, we acknowledge several limitations in this study. First, the sample size of clinical specimens (*n* = 4 paired HCC tissues) used for Western blot validation was relatively small, which may limit the generalizability of our findings to broader HCC populations. Future studies should validate CAPG expression patterns in larger, multi‐centre clinical cohorts. Second, although our xenograft models consistently demonstrated that CAPG knockdown enhanced Dox sensitivity, variations in tumour microenvironment, immune infiltration, and metabolic heterogeneity across different animal batches could potentially influence the reproducibility of these results. To mitigate this variability, we standardised animal age, sex and housing conditions, and performed experiments in at least two independent cohorts. Nonetheless, future preclinical studies should consider syngeneic or genetically engineered mouse models to better recapitulate human HCC heterogeneity.

## Author Contributions

**Yue Shang:** conceptualization (equal), data curation (equal), formal analysis (equal), investigation (equal), methodology (equal), resources (equal), software (equal), supervision (equal), writing – original draft (equal). **Jun Zhang:** data curation (equal), formal analysis (equal), investigation (equal), project administration (equal), resources (equal), visualization (equal). **Tingting Liu:** conceptualization (equal), funding acquisition (equal), methodology (equal), supervision (equal), validation (equal), visualization (equal), writing – review and editing (equal).

## Ethics Statement

All the experimental procedures were authorised by the Committees of Animal Ethics and Experimental Safety of The Second Affiliated Hospital of Dalian Medical University.

## Consent

All authors agree to publish.

## Conflicts of Interest

The authors declare no conflicts of interest.

## Supporting information


**Figure S1:** Kaplan–Meier survival analysis demonstrating the prognostic value of CAPG expression in different subgroups of HCC patients. High CAPG expression is associated with a poor prognosis in subgroups of race (White) (A), gender (Male) (B), age (> 60) (C), tumour status: with tumour (D), pathologic stage (T1&T2) (E), pathologic N stage: N0 (G), pathologic M stage: M0(H), pathologic stage: Stage I (I), pathologic stage: Stage III (J), histologic grade: G2(K) and vascular invasion: Yes (L). Overall survival hazard ratios (HR) and corresponding *p*‐values are provided for each subgroup.

## Data Availability

The authors have nothing to report.
