# Supplementary figures and images for: CAPG Regulates Doxorubicin Resistance in Hepatocellular Carcinoma Cells via TGFB1/Smad/Nrf2 Signalling Pathway
Source: J Cell Mol Med. 2025 Sep 22;29(18):e70847. doi: 10.1111/jcmm.70847 (PMC12453004; doi:10.1111/jcmm.70847)

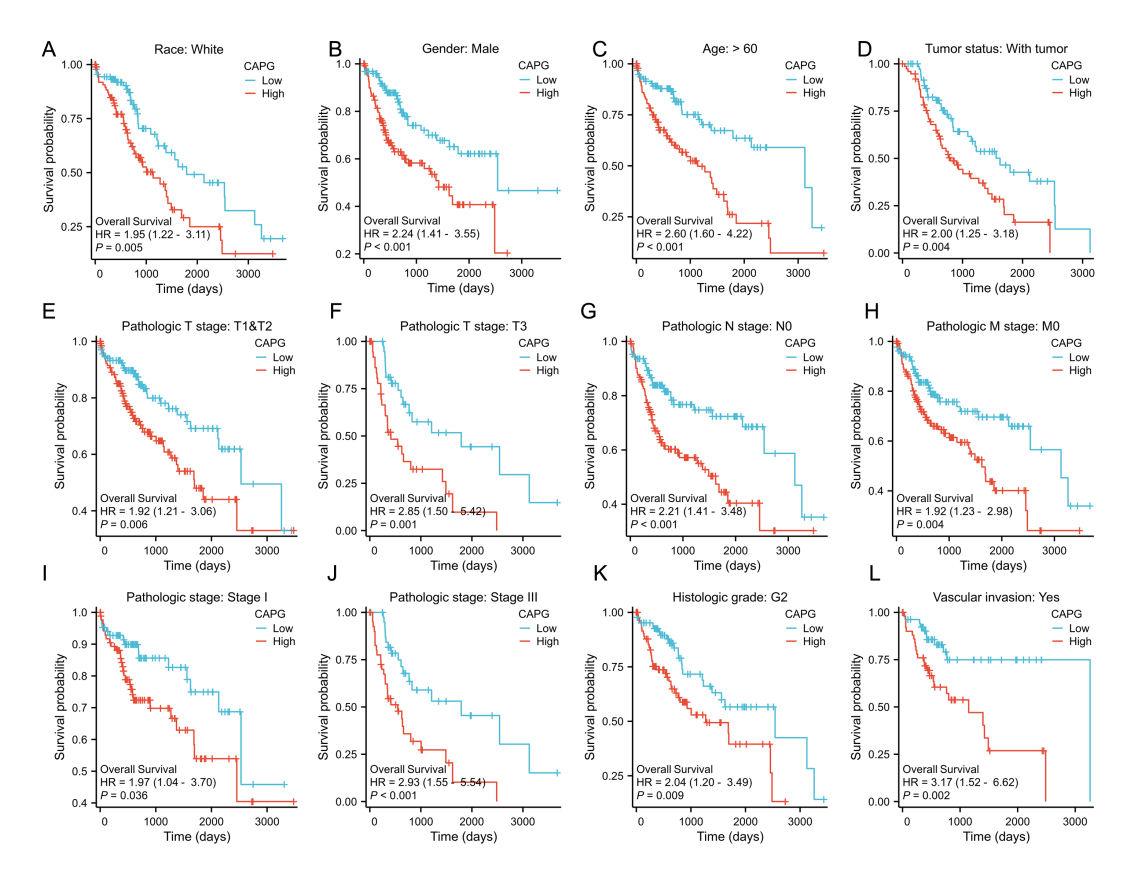

Supplement: Supplementary file 1 — Figure S1: Kaplan–Meier survival analysis demonstrating the prognostic value of CAPG expression in different subgroups of HCC patients. High CAPG expression is associated with a poor prognosis in subgroups of race (White) (A), gender (Male) (B), age (> 60) (C), tumour status: with tumour (D), pathologic stage (T1&T2) (E), pathologic N stage: N0 (G), pathologic M stage: M0(H), pathologic stage: Stage I (I), pathologic stage: Stage III (J), histologic grade: G2(K) and vascular invasion: Yes (L). Overall survival hazard ratios (HR) and corresponding p‐values are provided for each subgroup. [file JCMM-29-e70847-s001.tif]
